# Innovation in Digital Health Interventions for Frailty and Sarcopenia

**DOI:** 10.3390/jcm12062341

**Published:** 2023-03-17

**Authors:** Yuki Kato, Ryota Sakamoto, Asuka Hori, Ryo Momosaki

**Affiliations:** 1 Department of Rehabilitation Medicine, Mie University Graduate School of Medicine, Tsu 514-8507, Japan; 2 Department of Medical Informatics, Mie University Hospital, Tsu 514-8507, Japan

Today, the challenges of an aging society are primarily seen in frailty, sarcopenia, and impaired functionality. The definition of frailty pertains to a decreased ability to withstand the stress caused by the decline in reserve capacity with age [1]. According to the reported research, up to 10% of older adults in local communities are considered frail [2], and reports indicate that medical expenses for frail older adults are five times higher than for the rest of the population [3].

Sarcopenia is characterized by a decline in skeletal muscle mass and function that occurs with age. Research has shown that 30% of older community-based adults are affected by sarcopenia [4]. Sarcopenia has been reported to increase the likelihood of hospitalization by 60% and the incidence of falls by 70% [5]. The underlying causes of frailty and sarcopenia are believed to include factors such as low activity, malnutrition, and social isolation. As a result, interventions for the treatment of frailty and sarcopenia should include exercise, nutritional care, and social support [6].

The prevalence of frailty and sarcopenia is high; however, the diagnosis and evaluation of these conditions are time consuming and involve complex assessment tools and algorithms [7]. Therefore, a comprehensive and automated approach for the assessment, diagnosis, and intervention of these conditions is needed. Moreover, with the ongoing COVID-19 pandemic, noncontact and manageable methods are urgently required [8].

Digital health interventions (DHIs) have recently been introduced in various areas of healthcare [9]. Examples of such digital technologies include video calls, smartphone applications, internet websites, games, robots, and sensors with wearable devices [10]. DHIs have been used to treat numerous diseases and patient groups. In cases of frailty and sarcopenia in older adults, studies suggest that DHIs can aid in automating and enhancing diagnosis and intervention. Various studies have emphasized the use of smartphone applications and other DHIs for diagnosing and assessing frailty and sarcopenia [11,12,13]. These are simpler and more cost-effective alternatives to traditional face-to-face questionnaires and medical examinations. However, their validity in comparison with conventional assessment methods has yet to be fully established. The absence of strict diagnostic criteria, particularly for frailty, makes validation more difficult [14].

Engaging in physical activity, such as resistance exercise, has been proven to effectively prevent and improve frailty and sarcopenia [15]. However, sustaining physical activity can often be challenging. Retention rates and compliance with at-home self-directed training are low compared with supervised exercise programs [16].

Delivering exercise programs online is more feasible and leads to higher compliance compared with supervised exercise programs at a gym or other location [17]. Older adults with sarcopenia demonstrated increased skeletal muscle mass when using telecommunication services such as videophones to participate in exercise programs from their homes [18]. Furthermore, studies have reported that exercise programs using games and applications have similar effects to traditional exercise programs [19]. Recent studies on exercise programs using virtual reality technology have proven to be effective for older adults with sarcopenia [20], and the use of DHIs for remote exercise programs is expected to become more widespread in the future. Older adults who are frail and sarcopenic are at high risk of malnutrition; hence, interventions to improve their nutritional status may be necessary. Studies have reported that Internet- and application-based dietary assessment and nutritional support are effective in improving the nutritional status of older adults with sarcopenia [21]. In addition to smartphones, smart appliances are also being developed, and smart refrigerators that can monitor contents and usage may more efficiently improve the nutritional status of older adults with sarcopenia [22].

Frail and sarcopenic older adults with less muscle strength are at a high risk of falling, and once they fall, they may become fearful and less physically active, which may further reduce their muscle strength [23]. Therefore, the detection and prevention of falls in frail and sarcopenic older adults is an important issue. Sensors and wearable devices have been reported to be useful in assessing gait and balance in frail and sarcopenic older adults [24]. These devices can predict fall risk and prevent falls. One study demonstrated the use of in-ear wearable devices, similar to Bluetooth earbuds, for assessing gait; their accuracy was comparable to that of devices worn on the legs or hips [25]. In-ear wearable devices may be more useful for gait training and fall prevention because they are relatively inexpensive and can be easily equipped with voice-assist functions. Additionally, technologies have been developed to detect falls using smartphone applications and in-ear wearable devices based on changes in the angle and speed of the device when a user falls [26,27]. As these technologies become more widely implemented, we may observe a decrease in DHI-associated fall-risks.

Social isolation is associated with frailty and sarcopenia, but studies have suggested that the use of mobile applications may improve isolation in older adults [28]. Although there is currently no research focusing on frail and sarcopenic older adults, mobile applications could be used as one of the social prescriptions for preventing and improving frailty and sarcopenia.

DHIs using smartphones, wearable devices, and other devices are expensive, and cost-related concerns may hinder their use [29]. Some older adults also face challenges in successfully using these devices due to a lack of experience and counterintuitive usability [22]. Several studies have reported positive impacts on the use of electronic devices, cognitive function, and well-being after educating older adults about digital technology [30]. Older adults who are less proficient in the use of digital technologies may benefit from education and the development of easier-to-use technologies.

DHIs have been reported to help diagnose and manage frailty and sarcopenia, increase physical activity, improve nutritional status, reduce the risk of falls, and may also have potential for improving social isolation in the future. DHIs will increase in importance as the number of older people comfortable with digital technology increases [31]. There is high heterogeneity in the definitions of frailty and intervention methods used in studies on DHIs for frailty and sarcopenia. Of the limited studies on sarcopenia, many of them are small; therefore, more research is needed in the future.

## References

[1] Morley J.E., Vellas B., van Kan G.A., Anker S.D., Bauer J.M., Bernabei R., Cesari M., Chumlea W.C., Doehner W., Evans J. (2013). Frailty consensus: A call to action. J. Am. Med. Dir. Assoc..

[2] Collard R.M., Boter H., Schoevers R.A., Oude Voshaar R.C. (2012). Prevalence of frailty in community-dwelling older persons: A systematic review. J. Am. Geriatr. Soc..

[3] Bock J.O., König H.H., Brenner H., Haefeli W.E., Quinzler R., Matschinger H., Saum K.U., Schöttker B., Heider D. (2016). Associations of frailty with health care costs–results of the ESTHER cohort study. BMC Health Serv. Res..

[4] Cruz-Jentoft A.J., Landi F., Schneider S.M., Zúñiga C., Arai H., Boirie Y., Chen L.K., Fielding R.A., Martin F.C., Michel J.P. (2014). Prevalence of and interventions for sarcopenia in ageing adults: A systematic review. Report of the International Sarcopenia Initiative (EWGSOP and IWGS). Age Ageing.

[5] Scott D., Seibel M., Cumming R., Naganathan V., Blyth F., Le Couteur D.G., Handelsman D.J., Waite L.M., Hirani V. (2017). Sarcopenic obesity and its temporal associations with changes in bone mineral density, incident falls, and fractures in older men: The concord health and ageing in men project. J. Bone Miner. Res..

[6] Dent E., Lien C., Lim W.S., Wong W.C., Wong C.H., Ng T.P., Woo J., Dong B., de la Vega S., Hua Poi P.J. (2017). The asia-pacific clinical practice guidelines for the management of frailty. J. Am. Med. Dir. Assoc..

[7] Cruz-Jentoft A.J., Bahat G., Bauer J., Boirie Y., Bruyère O., Cederholm T., Cooper C., Landi F., Rolland Y., Sayer A.A. (2019). Writing group for the European working group on sarcopenia in older people 2 (ewgsop2), and the extended group for ewgsop2 sarcopenia: Revised European consensus on definition and diagnosis. Age Ageing.

[8] von Humboldt S., Mendoza-Ruvalcaba N.M., Arias-Merino E.D., Costa A., Cabras E., Low G., Leal I. (2020). Smart technology and the meaning in life of older adults during the COVID-19 public health emergency period: A cross-cultural qualitative study. Int. Rev. Psychiatry.

[9] World Health Organization (2016). Monitoring and Evaluating Digital Health Interventions: A Practical Guide to Conducting Research and Assessment.

[10] Linn N., Goetzinger C., Regnaux J.P., Schmitz S., Dessenne C., Fagherazzi G., Aguayo G.A. (2021). Digital health interventions among people living with frailty: A scoping review. J. Am. Med. Dir. Assoc..

[11] Lera L., Angel B., Márquez C., Saguez R., Albala C. (2020). Software for the diagnosis of sarcopenia in community-dwelling older adults: Design and validation study. JMIR Med. Inform..

[12] Chang R., Low H., McDonald A., Park G., Song X. (2021). Web-based software applications for frailty assessment in older adults: A scoping review of current status with insights into future development. BMC Geriatr..

[13] Teixeira E., Bohn L., Guimarães J.P., Marques-Aleixo I. (2022). Portable digital monitoring system for sarcopenia screening and diagnosis. Geriatrics.

[14] Hii T.B.K., Lainchbury J.G., Bridgman P.G. (2015). Frailty in acute cardiology: Comparison of a quick clinical assessment against a validated frailty assessment tool. Heart Lung Circ..

[15] Bauman A., Merom D., Bull F.C., Buchner D.M., Fiatarone Singh M.A. (2016). Updating the evidence for physical activity: Summative reviews of the epidemiological evidence, prevalence, and interventions to promote “active aging”. Gerontologist.

[16] Winett R.A., Williams D.M., Davy B.M. (2009). Initiating and maintaining resistance training in older adults: A social cognitive theory-based approach. Br. J. Sports Med..

[17] Vikberg S., Björk S., Nordström A., Nordström P., Hult A. (2022). Feasibility of an online delivered, home-based resistance training program for older adults—A mixed methods approach. Home-based resistance training program for older adults. Front. Psychol..

[18] Hong J., Kim J., Kim S.W., Kong H.J. (2017). Effects of home-based tele-exercise on sarcopenia among community-dwelling elderly adults: Body composition and functional fitness. Exp. Gerontol..

[19] Hagedorn D.K., Holm E. (2010). Effects of traditional physical training and visual computer feedback training in frail elderly patients. A randomized intervention study. Eur. J. Phys. Rehabil. Med..

[20] Chen G.B., Lin C.W., Huang H.Y., Wu Y.J., Su H.T., Sun S.F., Tuan S.H. (2021). Using virtual reality-based rehabilitation in sarcopenic older adults in rural health care facilities—A quasi-experimental study. J. Aging Phys. Act..

[21] Wang Z., Xu X., Gao S., Wu C., Song Q., Shi Z., Su J., Zang J. (2022). Effects of internet-based nutrition and exercise interventions on the prevention and treatment of sarcopenia in the elderly. Nutrients.

[22] Muse E.D., Barrett P.M., Steinhubl S.R., Topol E.J. (2017). Towards a smart medical home. Lancet.

[23] Ellmers T.J., Delbaere K., Kal E.C. (2023). Frailty, falls and poor functional mobility predict new onset of activity restriction due to concerns about falling in older adults: A prospective 12-month cohort study. Eur. Geriatr. Med..

[24] Greene B.R., Doheny E.P., Kenny R.A., Caulfield B. (2014). Classification of frailty and falls history using a combination of sensor-based mobility assessments. Physiol. Meas..

[25] Jung C.K., Kim J., Rhim H.C. (2023). Validation of an ear-worn wearable gait analysis device. Sensors.

[26] Torres-Guzman R.A., Paulson M.R., Avila F.R., Maita K., Garcia J.P., Forte A.J., Maniaci M.J. (2023). Smartphones and threshold-based monitoring methods effectively detect falls remotely: A systematic review. Sensors.

[27] Guilbeault-Sauvé A., De Kelper B., Voix J. (2021). Man down situation detection using an in-ear inertial platform. Sensors.

[28] Stuart A., Yan R.J., Harkin L.J., Katz D., Stevenson C., Mehta V., Giles E., Talbot C., Gooch D., Bennasar M. (2023). Digital intervention in loneliness in older adults: Qualitative analysis of user studies. JMIR Form. Res..

[29] Patel M.S., Asch D.A., Volpp K.G. (2015). Wearable devices as facilitators, not drivers, of health behavior change. JAMA.

[30] Lee H., Lim J.A., Nam H.K. (2022). Effect of a digital literacy program on older adults’ digital social behavior: A quasi-experimental study. Int. J. Environ. Res. Public Health.

[31] Zhao Y.C., Zhao M., Song S. (2022). Online health information seeking behaviors among older adults: Systematic scoping review. J. Med. Internet Res..

